# Novel Strategies for the Biodegradation and Detoxification of Mycotoxins in Post-Harvest Grain

**DOI:** 10.3390/toxins15070445

**Published:** 2023-07-05

**Authors:** Lihong Zhao, Desheng Qi, Qiugang Ma

**Affiliations:** 1 State Key Laboratory of Animal Nutrition, College of Animal Science and Technology, China Agricultural University, Beijing 100193, China; zhaolihongcau@cau.edu.cn; 2 Department of Animal Nutrition and Feed Science, College of Animal Science and Technology, Huazhong Agricultural University, Wuhan 430070, China; qds@mail.hzau.edu.cn

Mycotoxins are toxic secondary metabolites produced by filamentous fungi belonging, in particular, to the *Aspergillus*, *Fusarium*, and *Penicillium* genera. Aflatoxins, zearalenone (ZEN), deoxynivalenol (DON), T-2 toxin, ochratoxin A (OTA), fumonisins, patulin, and ergot alkaloids are the most contaminating mycotoxins found in food and feed, posing potentially carcinogenic, mutagenic, teratogenic, cytotoxic, neurotoxic, nephrotoxic, estrogenic, and immunosuppressant effects on both humans and animals [1]. Moreover, the broad spectrum of contamination caused by mycotoxins affects not only the economy but also poses a threat to public health due to the wide range of effects caused by contamination, which attracts the attention of researchers to explore novel approaches to detoxify mycotoxin-contaminated food and feed. This Special Issue therefore summarizes various strategies for the detoxification of mycotoxins through post-harvest detoxification methods, which are divided into physical, chemical, biological, and other developing innovative strategies. The physical strategies used to mitigate mycotoxins include rapid and proper drying, post-harvest insect control, good storage conditions, inorganic and organic adsorbents, such as montmorillonite and yeast cell walls, and advanced oxidation technology, such as irradiation and cold plasma, which allow for the rapid degradation of mycotoxins [2]. Chemical approaches involve the use of ozone, electrolyzed oxidizing water, organic acids, and natural plant extracts, which are widely accepted as safe food additives in many countries [3]. Biological approaches are defined as the microbial and enzymatic degradation of mycotoxins into non-toxic or less toxic metabolites. The biodegradation of mycotoxins is an emerging and frequently studied research topic. Many microorganisms and enzymes have been reported to degrade various mycotoxins in recent times [4].

This Special Issue aims to gather contributions of original research or reviews related to novel strategies for the biodegradation and detoxification of mycotoxins. Topics of interest will include, in particular, novel mycotoxin-degrading microorganisms and enzymes, fermentation technology to reduce the mycotoxin content in cereal products, and studies on alleviating the mycotoxicosis of livestock through the addition of bioactive substances or mycotoxin biodegradation agents.

The first study included in this Special Issue developed sustainable strategies to counteract mycotoxin contamination and cowpea weevil in chickpea seeds during the post-harvest period. The results showed that O_3_ significantly decreased the incidence of *Penicillium* spp. (by an average of −50%, independent of the time of exposure) and reduced the contents of patulin and aflatoxins (−85 and −100% after 30 min of exposure, respectively). High N_2_ concentrations significantly reduced mycotoxin contamination (by an average of −94%) and induced pest mortality (at 100% after 5 days of exposure). These results confirm the promising potential of O_3_ and N_2_ in post-harvest conservation strategies for eliminating mycotoxin contamination [5]. Five essential oils (thymol, carvacrol, cinnamaldehyde, eugenol, and citral) were tested for their inhibition effects against *Aspergillus flavus* and aflatoxin B1 production in broth and feed. The results showed that cinnamaldehyde and citral have a positive synergistic effect and that both of them could inhibit at least 90% of the fungal growth and aflatoxin B1 production in broth and poultry feed; thus, they could be an alternative to control aflatoxin contamination in food and feed in future [6]. Li et al. revealed that the disruption of redox genes is involved in the mechanism of coumalic acid and geraniol against *Aspergillus flavus* spore germination, and essential oils have a significant inhibitory effect on germination rates and redox gene expression [7]. Another study from the same group explored the fungistatic effect and mechanism of thymol on *Fusarium graminearum*, with the results showing that thymol can effectively inhibit the growth and toxin production of *F. graminearum* and cause an extensive transcriptome response, and the gluconeogenesis/glycolysis pathway may be a potential and important way for thymol to affect the growth of *F. graminearum* hyphae and the production of DON simultaneously [8].

Second, this Special Issue places a specific emphasis on the microbial and enzymatic transformation of mycotoxins in post-harvest detoxification strategies. A new *Alcaligenes faecalis* ANSA176 with a strong OTA-detoxifying ability was isolated from donkey intestinal chyme, which could degrade 97.43% of 1 mg/mL OTA into OTα within 12 h at 37 °C. The study of laying hens fed an OTA-contaminated diet showed that ANSA176 supplementation in their diet inhibited or attenuated the immune injury and inflammation induced by OTA through efficiently degrading OTA in the animals’ intestinal tract [9]. A study was conducted to compare the potential ameliorative effects between probiotic *Bacillus subtilis* (ANSB010) and biodegradable *B. subtilis* (ANSB01G) on ZEN toxicosis in gilts. The results showed that the ZEN-contaminated diet had a harmful impact on the growth performance, plasma immune function, and hormone secretion of gilts. Although probiotic and biodegradable *B. subtilis* have similar antimicrobial capacities, only biodegradable *B. subtilis* could eliminate these negative effects through its degradation of ZEN in the intestinal tract of gilts [10]. A laccase-degrading aflatoxin B1 from *B. amyloliquefaciens* B10 was isolated, purified, and characterized by Xiong et al. Their results showed that purified laccase could degrade 79.3% of AFB1 within 36 h, and the mutation of the three key metal combined sites (H-87, C-132, and H-149) of B10 laccase resulted in the loss of AFB1-degrading activity, indicating that these three metal combined sites of B10 laccase play an essential role in the catalytic degradation of AFB1 [11]. Ery4 laccase, acetosyringone, ascorbic acid, and dehydroascorbic acid were applied to artificially contaminated corn for AFB1 reduction, which showed that AFB1 (0.1 µg/mL) was completely removed in vitro and reduced by 26% in corn [12]. Wang et al. discovered that a manganese peroxidase (MrMnP) from *Moniliophthora roreri* can efficiently degrade patulin. The recombinant MrMnP was able to completely remove 5 mg/L of pure patulin within 5 h. Moreover, up to 95% of the toxin was eliminated in simulated patulin-contaminated apple juice after 24 h. The study concluded that MrMnP can be used as an intriguing candidate useful in the enzymatic detoxification of patulin in food and beverages [13]. A novel bacterium *ketogulonicigenium vulgare* D3_3 isolated from the feces of tenebrio molitor larvae was able to efficiently degrade 50 mg/L of DON under a broad range of conditions. Furthermore, four PQQ-dependent alcohol dehydrogenases responsible for the oxidative detoxification of DON were identified from the genome of isolate D3_3. These findings suggest that bio-detoxification is a potential strategy to remit the toxicity of DON in animals [14].

Finally, some studies have focused on the toxicity of mycotoxins in cells and animals and the alleviating effects of some nutritional and enzymatic additives on some mycotoxins. Chao et al. demonstrated that AFB1 exposure impaired the proliferation of porcine alveolar macrophage 3D4/2 via the non-coding RNA-mediated pathway by whole-transcriptome analysis [15]. Wang et al. also revealed that AFB1 exposure caused pathological damage to ducklings’ livers, decreased enzymatic activity and glutathione content in the liver, and increased the serum enzyme activities of alanine aminotransferase, aspartate aminotransferase, alkaline phosphatase, and γ-glutamyl transpeptidase. Moreover, the study found that dietary epigallocatechin gallate and glutathione attenuate AFB1-induced acute liver injury in ducklings via mitochondria-mediated apoptosis and the Nrf2 signaling pathway [16]. In addition, glutamine can alleviate ZEN-induced apoptosis in IPEC-J2 cells via the PI3K/Akt signaling pathway [17]. Meanwhile, dietary catalase supplementation alleviates DON-induced oxidative stress and intestinal damage in broilers, which can be associated with its ability to improve the gut microbiota, aside from its direct oxygen radical-scavenging activity [18]. 
